# Autoimmune Polyglandular Syndrome Type 1: a case report

**DOI:** 10.1186/s12881-019-0870-3

**Published:** 2019-08-16

**Authors:** Sayed Mahmoud Sajjadi-Jazi, Akbar Soltani, Samaneh Enayati, Armita Kakavand Hamidi, Mahsa M. Amoli

**Affiliations:** 1 Endocrinology and Metabolism Research Center, Endocrinology and Metabolism Clinical Sciences Institute, Tehran University of Medical Sciences, Tehran, Iran; 2 Cell therapy and Regenerative Medicine Research Center, Endocrinology and Metabolism Molecular-cellular Sciences Institute, Tehran university of Medical Sciences, Tehran, Iran; 3 Metabolic Disorders Research Center, Endocrinology and Metabolism Molecular-Cellular Sciences Institute, Tehran University of Medical Sciences, Tehran, Iran

**Keywords:** Single nucleotide variation, APS-1, AIRE gene, Mutation, APECED

## Abstract

**Background:**

Mutations of the autoimmune regulator gene (AIRE), located on chromosome 21q22.3, are recognized as the cause of a rare monogenic organ-specific autoimmune disorder called autoimmune polyglandular syndrome type 1 (APS-1). Three major components of this syndrome include chronic mucocutaneous candidiasis (CMC), hypoparathyroidism, and adrenocortical failure.

**Case presentation:**

We report a 19-year-old girl, who was born in an Iranian Muslim family with a clinical diagnosis of APS-1. To identify the causative mutation, a direct sequencing of the entire AIRE gene sequence was performed by Sanger sequencing method.

Three distinct variants were discovered, including c.1095 + 2 T > A, c.1197 T > C (rs1800521) and c.1578 T > C (rs1133779), in intron 9, exons 10 and 14 of the AIRE gene, respectively.

**Conclusions:**

To the best of our knowledge, this is the first report of an Iranian Muslim APS-1 patient with combination of these variations. In addition, the effect of c.1095 + 2 T > A mutation on AIRE mRNA expression was reported for the first time. This study expands the diversity of variants that could cause APS-1. More genetic studies are required to determine the exact frequency of these variants and their diagnostic significance.

## Background

Autoimmune polyendocrinopathy syndrome type 1 (APS-1), also called autoimmune polyendocrinopathy-candidiasis–ectodermal dystrophy/dysplasia (APECED), is a rare autosomal recessive syndrome (OMIM 240300) with a small female preponderance [1, 2]. It occurs more frequently in certain populations, including Iranian Jews (1/9000), Finns (1/25,000), and Sardinians (1/14,400) [3]. APS-1 is characterized by immune dysregulation which leads to some endocrine and nonendocrine manifestations [2].

To define this syndrome, patients must have at least two of the three major components, i.e. a chronic mucocutaneous candidiasis (CMC), hypoparathyroidism, and autoimmune adrenal insufficiency [4, 5]. Other components of APS-1 include type 1 diabetes, autoimmune hepatitis, hypothyroidism, primary hypogonadism, pernicious anemia, alopecia, ectodermal dysplasia, malabsorption, and vitiligo [6].

The disease’s inheritance is in the Mendelian fashion [7]. The underlying genetic abnormality which causes APS-1 is a mutation in the Autoimmune Regulator (AIRE) gene mapped to chromosome 21q22.3 [8]. Fourteen exons of this gene encode a proline-rich protein with 545 amino acids [7]. This protein plays a role in the regulation of transcription [7]. There are distinctive subdomains in the AIRE protein structure, including a highly-conserved N-terminal homogeneously staining region (HSR) domain (exons 1 & 2), the nuclear localization signal (NLS) (exon 3), the putative DNA binding SAND domain (exons 5, 6 & 7), two plant homeodomain (PHD) type zinc fingers (PHD1: exons 8 & 9, PHD2: exons 11 & 12), the proline-rich region (PRR) (exon 10), and four LXXLL motifs [7, 9, 10]. To date, many different mutations (more than 115 mutations) [9] including small insertions, deletions, and single nucleotide substitutions [11] have been reported with the predominant gene mutation varying across different ethnic groups [12, 13]. It has been suggested that mutation in different regions of the AIRE gene has various results on the function of the AIRE protein with a currently poorly-described mechanism [7, 9, 14]. In this article, we report three AIRE variations in a patient with APS-1 from an Iranian Muslim family.

## Case presentation

### Patient

The patient was a 19-year-old girl who was born in an Iranian Muslim family. She was referred to our center (Shariati hospital, Tehran, Iran) due to her low back pain and multiple vertebral fragility fractures. Other important complaints of her were including primary amenorrhea, peri-oral, and acral paresthesia, blurred vision, and also foreign body sensation. She did not have any signs or symptoms of malabsorption.

#### Past medical history

Her past medical history was significant for hypocalcemia-induced generalized tonic-clonic seizures (three episodes), when she was at the age of 6, due to hypoparathyroidism. Consequently, she received calcitriol and calcium supplementation, but her compliance was poor. At the age of 7, she was diagnosed with alopecia areata. She was suffering from asthma at the same time, for which she used salbutamol and montelukast. Being short in stature at the age of 13.5 years old (height = 134 cm, standard deviation score (SDS) = − 3.8), she was evaluated whereby the growth hormone (GH) deficiency was revealed; however, due to financial problems, she could not afford the costs and therefore did not follow through the GH treatment. She had also been experiencing a history of CMC involving oral cavity and nails since the age of 14 years old. Her other complaint was dry eye symptoms for 2 years, and for this reason she have been receiving artificial tears. She had also taken a course of oral acyclovir with the diagnosis of herpes simplex keratopathy. She was not on a regular follow-up before being referred to our center.

#### Family history

**Fig. 1:**
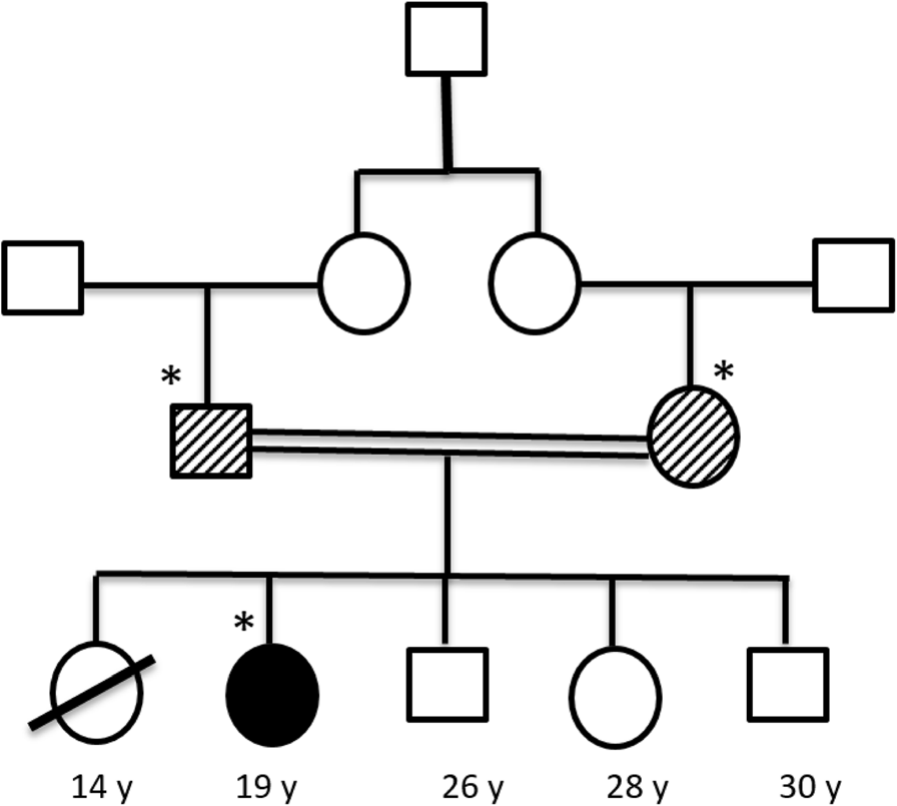
The patient’s pedigree chart. *Mutation screening was done for the patient and her parents

Her family history was remarkable for her parents’ consanguineous marriage, and also her sister death at the age of 14 years old, with no definitive diagnosis. Her sister had a history of blindness due to endophthalmitis and her symptoms before death were weakness, loss of appetite, fever, nauseas, and vomiting. The other patient’s siblings were healthy (two 30 and 26-year-old brothers and a 28-year-old sister). The patient’s pedigree chart is illustrated in Fig. 1.

#### Managements in our center

Due to hypoparathyroidism (calcium = 6.6 mg/dl, Phosphorus = 7.8 mg/dl, iPTH = < 8 pg/ml), an adequate dose of calcium (3 g/day elemental calcium in divided doses) and calcitriol (0.5 micrograms daily) supplementation were initiated, with sevelamer (800 mg three times per day) being later added, in regard with the inadequate phosphorus control. The patient complaint of primary amenorrhea and her physical examination revealed delayed puberty (Tanner stage 3 for breast development and stage 2 for pubic hair). With the diagnosis of premature ovarian failure (FSH = 64.5 IU/l, LH = 45.6 IU/l, prolactin: 35 ng/ml), hormone replacement therapy was initiated (0.625 mg conjugated equine estrogens for 21 days and 5 mg medroxyprogesterone acetate for 10 days). Levothyroxine (50 micrograms daily), was also added due to subclinical hypothyroidism (TSH = 9.3 μIU/ml, TT4 = 9.8 μgr/dl, TT3 = 166 ng/dl, antithyroid peroxidase antibody = positive). Adrenal insufficiency was ruled out by the Cosyntropin test (baseline ACTH = 60 pg/ml, baseline cortisol = 11 μgr/dl, peak cortisol = > 50 μgr/dl; note that adrenal autoantibodies were not checked due to the absence of these tests in Iran). Because of short stature (height = 137 cm, SDS = − 4), GH test was performed, which confirms GH deficiency (IGF1 = 66 ng/ml [NL: 198–551], clonidine test: peak GH = 2.8 mIU/l). GH treatment was offered, however, she did not take the GH treatment due to the financial problems. Her liver enzymes were elevated: ALT = 108 IU/l (NL: Up to 32), AST = 84 IU/l (NL: Up to 31), and ALP = 409 IU/l (NL: 64–306). The hepatitis viral markers were negative and serum protein electrophoresis demonstrated an elevation of serum IgG. Liver biopsy was performed, which indicated a chronic hepatitis (modified histology activity index grading: 9 [15], modified histology activity index staging: 3 [15]), compatible to autoimmune hepatitis. The patient was then referred to a gastroenterologist for further work-up and treatment. The other laboratory tests were normal, ruling out celiac disease (anti-endomysial antibody = negative), diabetes mellitus, and B12 deficiency.

Ophthalmologic consultation was done, because of foreign body sensation and blurred vision, which showed dry eyes and keratopathy due to a limbal stem cell deficiency. Cyclosporine eye drop, vitamin A eye ointment, fluorometholone eye drop, and artificial tears were initiated, and later inferior punctum closure was performed for the patient in order to improve her dry eyes.

Multiple vertebral fractures, which results in spine deformity, were evident in her spine x-ray. Bone mineral densitometry (BMD) was performed which revealed osteoporosis; height adjusted lumbar spine, femoral neck, and total hip BMD Z-Score were − 5.80, − 4.07, and − 2.80, respectively [16]. Other imaging studies including brain computed tomography (CT) and abdominopelvic ultrasound were normal, except for an infantile uterus and small ovaries in ultrasound.

The patient’s clinical findings are summarized in Table 1. With the diagnosis of APS-1, genetic testing was performed, which indicated the AIRE gene variations.

**Table 1:** Patient’s clinical findings

Diagnosis	Age of presentation/diagnosis (year)	Presentation	Treatments
Hypoparathyroidism	6	Generalized tonic-clonic seizures	Calcitriol and calcium, later sevelamer was added
Alopecia areata	7	Hair loss	Intralesional corticosteroid injections
Asthma	7	Dyspnea	Salbutamol and montelukast
GH deficiency	13.5	Short stature	No treatment
Chronic mucocutaneous candidiasis	14	Oral cavity and nails candidiasis	Course of fluconazole
APS1-associated keratopathy	17	Dry eye symptoms	Artificial tears, later cyclosporine eye drop, vitamin A eye ointment and fluorometholone eye drop were added and finally inferior punctum closure was performed
Osteoporosis, vertebral fragility fractures	19	Low back pain	Hormone replacement therapy
Premature ovarian failure	19	Primary amenorrhea	Hormone replacement therapy
Subclinical hypothyroidism	19	Abnormal thyroid function tests	Levothyroxine
Autoimmune hepatitis	19	Abnormal liver function tests	Referred to a gastroenterologist
Dental enamel hypoplasia	–	Dental decay	Dental care

### Mutation analysis

The DNA was extracted from peripheral blood lymphocyte, using the phenol-chloroform method. Primers encompassing the AIRE gene region were previously described [17]. Primer sequences and polymerase chain reaction (PCR) conditions for the amplification of the AIRE gene region are listed in Table 2. The PCR was performed by thermal cycler (BIO RAD, MJ mini thermal cycler, model: PTC-1148, made in Singapore) and the PCR protocol was set as follows: 5 min for the initial denaturation at 95 °C, 35 cycles of amplification (each cycle consisted of 30 s at 95 °C, 30 s of annealing at a specific temperature for each of the primer pairs (Table 2) and 30 s of extension at 72 °C), followed by the final extension at 72 °C for 5 min. Direct sequencing (Sanger sequencing method) was used for identifying the probable variation of each exon-intron. Chromas software (version 2.6.2) and National Center for Biotechnology Information (NCBI) gene databases (https://www.ncbi.nlm.nih.gov/) such as Basic Local Alignment Search Tool (BLAST), Clinical Variation (ClinVar), and Single Nucleotide Polymorphism database (dbSNP) were used for the sequence analysis.

**Table 2:** Primer sequences and PCR conditions for the amplification of AIRE gene regions

Exon	Primer sequence (5′ → 3′)	Product size (bp)	Annealing temperature (°C)
1	F: AAGCGAGGGGCTGCCAGTGTC R: GGGACTATCCCTGGCTCACAG	258	67–60 touch down
2	F: TCCACCACAAGCCGAGGAGAT R: AGCTGGGCTGAGCAGGTGACA	389	67–60 touch down
3	F: CTGAGGTTGGGACCCTGCTCC R: CTGGAGACCCTGGCTGGCTTC	231	68
4	F: AGAAACCAGAGCCCGGCAAAGG R: AATGACACACCAGGCCAGCACG	338	63
5	F: GCCCAGTGCTGCCTGCTTCTG R: CCATCTTGGAGCCTGGGTCTC	256	64
6	F: TGCAGGCTGTGGGAACTCCAC R: GGGGCATCAAGAGCCAGGCTC	305	65.4
7	F: CATGTGCACCCTCGCTGCTGA R: AGAAAAAGAGCTGTACCCTGTGG	278	65.2
8	F: CACCCCAGCCCAGTCTGCATG R: CTTCAGGGTCAGTGGGTGGAG	230	68
9	F: CTGTCACCCGCTGTCTTGTTC R: GTGGCCATGTGGACAGGAGG	205	63
10	F: CCCAGCAGTCACTGACTCCTG R: CGTAGGTCCTGGGCTCCTTGA	311	68
11	F: CTCGGGTTCGGGTTCAGCTAC R: TGTGGGTGTGGGTTCAGGCCT	233	72–65 touch down
12	F: CATACCCCGGAGGTGGCACTC R: CAGCACCGGCATGCATGGAGG	205	68
13	F: AGTGGGACTCCTTGCTGGTTCC R: AGGGACAGCCTGAGTTTCCACG	487	67
14	F: ATGGCCATGATTCTGTGGCTG R: CTCAGCACTCTCTCATCAGAG	183	68

*F* forward, *R* reverse, *bp* base pair

### Gene expression analysis

Peripheral blood mononuclear cells (PBMCs) were isolated from whole blood samples by the density gradient centrifugation method via Ficoll-Paque PLUS (GE life science). Whole Ribonucleic Acids (RNAs) were extracted from the PBMCs using Trizol reagent. The total messenger RNAs (mRNAs) were reversely transcribed into complementary DNAs (cDNAs) by random primers (K1622, Thermo scientific). Real-time PCR was performed on the LightCycler® 96 System (Roche) using SYBR Green and the specific primer for AIRE mRNA. The hypoxanthine guanine phosphoribosyl transferase (HPRT) was used as the internal control. The sequences of primers for real-time PCR are reported in Table 3. All samples were run in triplicates. The PCR reaction was performed by 10 μl fast start essential SYBR Green Master (Roche), 0.8 μl forward primer, 0.8 μl reverse primer, 6 μl cDNA (1/10 diluted), and 2.4 μl PCR grade water, with an initial denaturation step of 10s at 95 °C, 40 cycles at 95 °C for 5 s, and 60 °C for 30s. The fold changes were determined as 2^-ΔΔCT^.

**Table 3:** Primer sequences and PCR conditions for the real-time PCR of AIRE mRNA

mRNA name	Primer sequence (5′ → 3′)	Product size (bp)	Annealing temperature (°C)
AIRE	F: CACGACTCTTGTCTACAAGC R: AGGAGCCAGGTTCTGCT	124	60
HPRT	F: CCTGGCGTCGTGATTAGTGAT R: AGACGTTCAGTCCTGTCCATAA	131	60

*F* forward, *R* reverse, *bp* base pair

### Mutation and gene expression analysis results

**Fig. 2:**
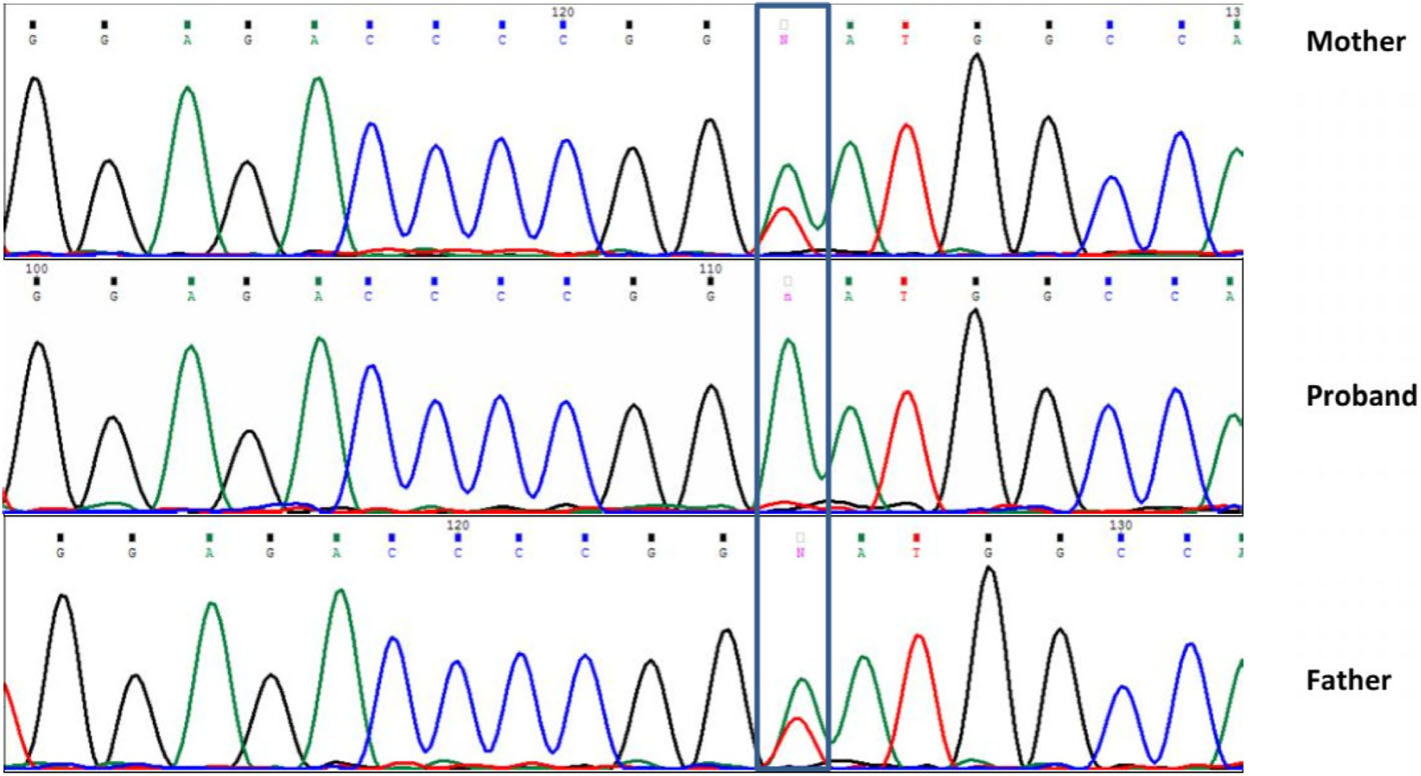
Sanger sequencing chromatogram of AIRE gene (part of intron 9), which indicated a homozygous and heterozygous c.1095 + 2 T > A mutation in the proband and her parents, respectivley

Sequencing the whole coding region and the exon-intron borders of the AIRE gene demonstrated three variants in the patient: a homozygous mutation in intron 9 (c.1095 + 2 T > A); a homozygous single nucleotide synonymous variant in exon 10 (c.1197 T > C, rs1800521); and a homozygous single nucleotide synonymous variant in exon 14 (c.1578 T > C, rs1133779). In addition, carrier screening was carried out in both parents, which confirmed heterozygous c.1095 + 2 T > A mutation (Fig. 2).

**Fig. 3:**
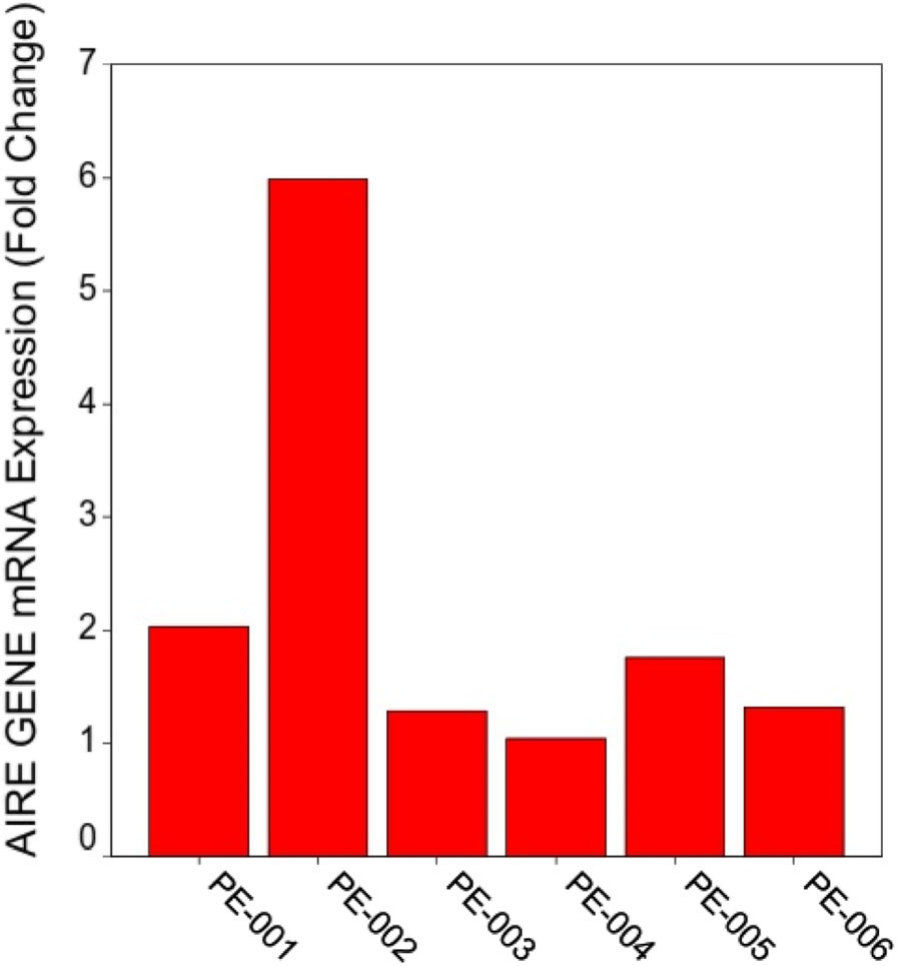
Expression levels of AIRE gene mRNA: PE-002 column belongs to the case with homozygous mutation in intron 9 (c.1095 + 2 T > A); PE-001 and PE-005 represent the expression levels in patient’s parents with heterozygous mutation in intron 9 (c.1095 + 2 T > A); other columns show the expression level of AIRE in siblings (mutations were not checked)

The expression levels of the AIRE mRNA in PBMCs of the patient, her parents and her three siblings are shown in Fig. 3. According to this figure, compared to other family members with no sign of disease, the AIRE mRNA has been upregulated in the patient with a homozygous intron 9 mutation.

In silico analysis using Human Splicing Finder (http://www.umd.be/HSF/) [18] has suggested that c.1095 + 2 T > A variation may result in alteration of an exonic splicing enhancer (ESE) site causing potential alteration of splicing.

## Discussion and conclusions

To date, many APS-1-causing mutations have been identified in the AIRE gene across different ethnic groups [9]. The Iranian Jewish population is well-known for carrying a founder AIRE gene mutation (Y85C) which causes a tyrosine to a cysteine change in the HSR domain [14]. Other reported AIRE mutations in the Iranian population have been R139X, R257X, K50NfsX168, and L323SfsX51 [12].

In line with the recessive mode of the inheritance of APS-1, most mutations of the AIRE gene are homozygous or compound heterozygous [7, 9, 19, 20]. Pathogenic mutations spread over the entire coding sequence of the AIRE gene where at least four mutational hot spots including exons 2, 6, 8, and 10 have been reported [11]. The proband in our study had one silent polymorphism in the hot spot exon 10 and one silent polymorphism in exon 14. Moreover, the promoter region or the intronic sequence mutation with an impact on the transcription and/or RNA splicing is possible [11, 12]. Our proband had one intronic mutation with a probable RNA splicing effect.

Two variants, c.1197 T > C and c.1578 T > C, which were observed in our patient, were reported for the first time in 1998 [21–23]. These two synonymous variants, one in exon 10 (proline-rich region) and the other in exon 14 (near one of the four interspersed LXXLL motifs) do not change the amino acid type at the protein level and are unlikely to have functional implications. In other words, the substitution of the T nucleotide in position 1197 to C (c.1197 T > C) does not change alanine amino acid in position 399 (p.Ala399=); similarly, the substitution of the T nucleotide in position 1578 to C (c.1578 T > C) does not alter aspartate amino acid in position 526 of the AIRE protein (p.Asp526=). Cetani et al. reported a 38-year-old Italian APECED woman who carried a G228 W mutation and three common silent heterozygous polymorphisms (588 C/T, S196S; 1197 T/C, A399A; and 1578 T/C D526D) [11]. Sun et al. also presented a 15-year-old Chinese APS-1 patient with c.57 T > C, c.588C > T, c.834C > G, c.1197 T > C, and c.1578 T > C SNPs [24]. These findings which are in line with our results highlight the fact that the two SNPs c.1197 T > C and c.1578 T > C are possible to occur at the same time.

The substitution of the T nucleotide in the second nucleotide of intron 9 to A (c.1095 + 2 T > A) is located in a region between PHD1 and PRR. This mutation occurs in the conserved splice donor sequence and leads to a change in the wild type donor site, affecting splicing [12]. Recently, Seifi-Alan et al. reported an Iranian Muslim family with the same mutation in intron 9 (c.1095 + 2 T > A) in which the parents were both heterozygous for this mutation. The 9-year-old proband in this report suffered from APS-1 (CMC, Addison’s disease, hypoparathyroidism, and hypothyroidism) [12]. The proband in our study had the same phenotype (with the exception of late onset CMC and the lack of Addison’s disease in our case), suggesting that c.1095 + 2 T > A may be regarded as a founder mutation in Iranian population, which cause APS-1 development. The late onset CMC and the lack of adrenal insufficiency in our case may be due to the fact that even patients with the same AIRE mutations present with a wide range of clinical manifestations and different courses of the disease [13]. According to Perheentupa et al., who followed up 91 APS1 patients up to 50 years, the initial manifestation of APS1 appeared at age 0.2–18 years. Among the main signs of APS1, CMC, hypoparathyroidism, and adrenal insufficiency were the presenting features in 60, 32, and 5% of patients, respectively. The CMC, hypoparathyroidism, and adrenal insufficiency affected 100, 87, and 81% of APS1 patients respectively by the time they were 40 years old [25]. The presenting feature in our case was hypoparathyroidism (age of 6) and later at age of 14, CMC was detected which is congruent with some cases of Perheentupa report. Our patient did not have adrenal insufficiency. Nevertheless, as we did not check anti-adrenal autoantibodies, the adrenal autoimmunity was not excluded. The vertebral fragility fractures in our case was justified by a combination of GH and estrogen deficiency.

Although the main expression of AIRE occurs in the medullary tissue of thymus [23], the dendritic cells of the blood also express this gene [26, 27]. Therefore, we examined the mRNA level of this gene in the PBMCs to test the effect of this splicing site mutation on the expression level. Our study indicated an up-regulation of AIRE expression in the patient with a homozygous c.1095 + 2 T > A mutation in comparison to patient’s parents with heterozygous mutation and other siblings (Fig. 3). The AIRE mRNA level was not reported in Seifi-Alan et al.’s report [12], and as a result, we could not compare this finding to pervious results. However, Björses et al. reported 13 Iranian Jews with APS-1 who had an A374G (Y85C) mutation in exon 2 with a transcriptional activity of 115% of the wild-type AIRE. Transcriptional activity of the Y85C mutation was in contrast to other mutations with low or no transcriptional activity reported by Björses et al. [7]. The mechanism suggested for this finding is that while the Y85C mutant AIRE protein has a correct length, it has a significantly shorter half-life and as a result, it rapidly deteriorates [14]. A similar mechanism may explain a higher mutant AIRE mRNA expression level we found here. Further studies are required to examine the effect of this intronic mutation (c.1095 + 2 T > A) on the function and stability of the AIRE protein and its cellular consequences. The expression level of the AIRE mRNA was assessed in PBMCs in our study, not at the site of its actual transcriptional activity located at thymus. Therefore, further investigations in the thymic tissue are required to confirm our results.

In conclusion, we identified three previously reported AIRE gene variations in an Iranian Muslim APS-1 patient. Furthermore, the association between c.1095 + 2 T > A mutation and APS-1 disease was confirmed in this report. Finally, AIRE gene expression analysis was performed, which indicated the functional impact of this variation for the first time. This study expands the diversity of variants that could cause APS-1. In addition to diagnostic utility, this information will help us better understand the pathophysiology of APS-1. More genetic studies are required to determine the frequency of these variants and their diagnostic credibility.

## Data Availability

Not applicable.

## Abbreviations

ACTH: Adrenocorticotropic Hormone
AIRE: Autoimmune Regulator gene
ALP: Alkaline Phosphatase
ALT: Alanine Aminotransferase
APECED: Autoimmune Polyendocrinopathy-Candidiasis–Ectodermal Dystrophy/Dysplasia
APS-1: Autoimmune Polyglandular Syndrome type 1
AST: Aspartate Aminotransferase
BLAST: Basic Local Alignment Search Tool
BMD: Bone Mineral Densitometry
cDNAs: Complementary DNAs
ClinVar: Clinical Variation
CMC: Chronic Mucocutaneous Candidiasis
CT: Computed Tomography
dbSNP: Single Nucleotide Polymorphism database
ESE: Exonic Splicing Enhancer
FSH: Follicle stimulating hormone
GH: Growth Hormone
HPRT: Hypoxanthine Guanine Phosphoribosyl Transferase
HSR: Homogeneously Staining Region
IGF1: Insulin like growth factor 1
IgG: Immunoglobulin G
iPTH: Intact Parathyroid hormone
LH: Luteinizing Hormone
mRNAs: Messenger RNAs
NCBI: National Center for Biotechnology Information
NLS: Nuclear Localization Signal
PBMCs: Peripheral Blood Mononuclear Cells
PCR: Polymerase Chain Reaction
PHD: Plant Homeodomain
PRR: Proline-Rich Region
RNAs: Ribonucleic Acids
SDS: Standard Deviation Score
TSH: Thyroid Stimulating Hormone
TT3: Total Thyroxine
TT4: Total Triiodothyronine
